# Circulating adipokine concentrations and the risk of venous thromboembolism: A Mendelian randomization and mediation analysis

**DOI:** 10.3389/fgene.2023.1113111

**Published:** 2023-03-28

**Authors:** Weizhong Xiao, Jian Li, Tianyuyi Feng, Long Jin

**Affiliations:** ^1^ Department of Interventional Radiology, Beijing Friendship Hospital, Capital Medical University, Beijing, China; ^2^ The Department of Radiology of the Fifth People’s Hospital of Shanghai, Fudan University, Shanghai, China

**Keywords:** Mendelian randomization analysis, venous thromboembolism, deep vein thrombosis, pulmonary embolism, adipokine, mediation analysis, obesity

## Abstract

**Background:** Previous observational studies have suggested that circulating adipokine concentrations are related to a greater risk of venous thromboembolism (VTE). However, it remained unclear whether these observations reflect causality.

**Objective:** This study aimed to investigate the causal relationship between circulating adipokine concentrations (including adiponectin, leptin, PAI-1, MCP-1, leptin receptor, and RETN) and the risk of VTE and its subtypes (DVT and PE) and to determine whether circulating adipokine concentrations are a mediator of venous thromboembolic events in obese patients.

**Methods:** We used Mendelian randomization (MR) analyses to determine the effects of the body mass index (BMI), adiponectin, leptin, PAI-1, MCP-1, leptin receptor, and RETN levels on VTE, DVT, and PE in a cohort of 11,288 VTE cases, 5,632 DVT cases, 5,130 PE cases, and 254,771 controls. We then assessed the proportion of the effect of obesity on VTE, DVT, and PE explained by circulating leptin levels.

**Result:** Genetically predicted higher BMI was related to increased VTE (OR = 1.45, *p* < 0.001), DVT (OR = 1.63, *p* < 0.001), and PE (OR = 1.37, *p* < 0.001) risk, and higher circulating leptin levels increase odds of VTE (OR = 1.96, q < 0.001), DVT (OR = 2.52, q < 0.001), and PE (OR = 2.26, q = 0.005). In addition, we found that the causal effect between elevated serum adiponectin and the decreased risk of VTE (OR = 0.85, *p* = 0.013, q = 0.053) and PE (OR = 0.81, *p* = 0.032, q = 0.083) and between MCP-1 and the reduced risk of VTE (OR = 0.88, *p* = 0.048, q = 0.143) is no longer significant after FDR adjustment. In MR mediation analysis, the mediation effect of circulating leptin levels in the causal pathway from BMI to PE was estimated to be 1.28 (0.95–1.71, *p* = 0.10), accounting for 39.14% of the total effect.

**Conclusion:** The circulating leptin level is a risk factor for VTE, DVT, and PE, but it might be a potential mediator of BMI on the risk of PE, and thus, interventions on the circulating leptin level in obesity might reduce the risk of PE. Adiponectin is a potential protective factor for both VTE and PE.

## Introduction

Venous thromboembolism (VTE) is a chronic cardiovascular disease that mainly consists of deep vein thrombosis (DVT) and pulmonary embolism (PE). Each year, VTE affects roughly 10 million individuals of all races globally and significantly contributes to the burden of illness worldwide (ISTH Steering Committee for World Thrombosis Day, 2014). Medical expenditure related to venous thromboembolism is approximately worth €1.5 to €3 billion every year in Europe and $7 to $10 billion in the United States (Barco et al., 2016; Grosse et al., 2016). After a heart attack or stroke, acute venous thromboembolism is the third most prevalent acute cardiovascular illness. It has an annual incidence of roughly 1–2/1,000, rising exponentially with age in both men and women. Meanwhile, VTE is four times more prevalent in high-income nations than in low-income nations (Tagalakis et al., 2013; ISTH Steering Committee for World Thrombosis Day, 2014; Heit, 2015).

High body mass index (BMI) has been identified as a risk factor of VTE in several major observational studies (Borch et al., 2010; Cushman et al., 2016; Klarin et al., 2017). According to the 2016 ISTH SSC guideline and its 2021 update, oral anticoagulants are recommended for VTE prophylaxis in patients with a BMI greater than 40 kg/m^2^ (Martin et al., 2016; Martin et al., 2021). Recent Mendelian randomization and observational research have further indicated the causal association between obesity and an increased incidence of VTE (Lindström et al., 2017; Kim et al., 2021; Yuan et al., 2021).

The molecular processes associating adiposity and the development of VTE have not yet been elucidated, which may be relevant to insulin resistance, adipocyte hypertrophy, and increased production of inflammatory adipocytokines and free fatty acids (Yang et al., 2012). As a highly active metabolic and endocrine organ, adipose tissue is now recognized to express and secrete a variety of adipokines regulating numerous metabolic processes and inflammatory responses inside the body (Halberg et al., 2008; Ouchi et al., 2011; Lehr et al., 2012; Raucci et al., 2013). Previous research has connected adipokines to various obesity-related diseases, with greater adiponectin levels deemed cardioprotective and higher leptin and resistin levels usually seen as detrimental to cardiovascular health. However, the causal association between adipokines and VTE is not well-established, and previous observational studies produced inconsistent results.

Conventional observational epidemiological studies are susceptible to residual confounding and reverse causality and are consequently incapable of drawing causal inferences. Mendelian randomization (MR) is an epidemiological methodology based on germline genetic data that increases causal inference in association by employing genetic variations as the exposed instrumental variables (Burgess and Thompson, 2015). As a result of the random assignment of alleles from parents to children during inheritance, Mendelian randomization prevents interference from potential confounding variables and reverse causal effects (Burgess and Thompson, 2015).

We used a two-sample MR framework to examine the association of six adipokines (adiponectin, leptin, PAI-1, MCP-1, leptin receptor, and RETN) with the risk of VTE and its two subtypes (DVT and PE) using genetic variants associated with adipokine concentrations from published genome-wide association studies (GWAS). In addition, we used the mediation analysis method based on multivariable Mendelian randomization (MVMR) to explore the proportion mediated by adipokines for the effect between BMI and VTE, DVT, and PE.

## Materials and methods

With publicly accessible datasets that yielded genome-wide association results for BMI, adipokines, and outcomes (VTE, DVT, and PE), we conducted two-step two-sample MR. The two-sample MR refers to the use of various datasets (samples) to determine the relationship between genes and risk factors (like BMI and adipokines) and outcomes (like VTE). We used two-step MR to first examine the effects of BMI and adipokines on outcomes. The first step was to determine the causal effect of BMI and adipokines on outcomes. In the second step, we figured out the causal effect between BMI and the significant adipokines (q < 0.05) found in the first step.

### Data sources and instrumental variables selection

#### Genetic instrumental variables selection for adipokines

The genetic association for adipokines was retrieved from a previously published genome-wide association study (GWAS). Single-nucleotide polymorphisms (SNPs) were identified to be associated with exposures with *p*-values at the genome-wide significance level (*p* < 5 × 10^−8^). SNPs with *R* ^2^ > 0.01 and within 5,000 kb distance were identified to be in linkage disequilibrium and were excluded from the study. In total, six adipokines were chosen for this study.

The summary-level data related to the serum adiponectin level from a GWAS in 39,883 individuals of European ancestry, and a total of 12 SNPs were selected as instrumental variables (IVs) (Dastani et al., 2012). The summary-level data related to circulating leptin levels from a GWAS meta-analysis which include 57,232 adults (≥18 years old), of whom 50,321 were of European ancestry, 4,387 of African ancestry, 2,036 East East Asian ancestry, and 488 of Hispanic ancestry, adjusting for age, genome-wide principal components, and any study-specific covariates (e.g., study center). Four SNPs associated with circulating leptin level IVs (Yaghootkar et al., 2020). Data on DNA methylation estimations of plasminogen activator inhibitor-1 (PAI-1) levels at the summary level were taken from a GWAS that included 34,448 people of European ancestry and were adjusted for age (McCartney et al., 2021). We selected a total of four SNPs as IVs. Data on plasma levels of monocyte chemoattractant protein-1 (MCP-1) and resistin (RETN) at the summary level were taken from the SCALLOP CVD1 meta-analysis that included up to 21,758 European ancestries (Folkersen et al., 2020). As IVs, we utilized six SNPs for MCP-1 and 12 SNPs for RETN. For the leptin receptor, we obtained the summary-level data from the IEU-Open GWAS project (GWAS ID: prot-a-1724), which included 3,301 individuals of European descent, adjusted for age, sex, duration between drawing and processing the blood (binary, ≤ 1 day/>1 day), and the first three principal components of ancestry from multi-dimensional scaling in a linear regression, and we used three SNPs as IVs (Sun et al., 2018).

#### Genetic instrumental variables selection for BMI

The genetic association for BMI was retrieved from a GWAS study reported by the genetic investigation of anthropometric trait (GIANT) consortium (Locke et al., 2015). This study included association results for up to 339,224 individuals from 125 studies, 82 with GWAS results (n = 236,231) and 43 with results from metabochip (n = 103,047). A total of 322,154 people of European ancestry and 17,072 people of non-European ancestry were analyzed. We adopted 322,154 GWAS summary-level data of European origin for the study to guarantee the validity of the findings. Single-nucleotide polymorphisms (SNPs) associated with the exposures were obtained with *p*-values at a genome-wide significance level (*p* < 5 × 10^–8)^. SNPs with *R* ^2^ > 0.01 and within 5,000 kb distance were identified to be in linkage disequilibrium and were subsequently excluded from the analysis. Eventually, 84 SNPs were extracted for BMI and were used as instrumental variables (IVs).

#### Outcome

##### VTE and subtypes

We obtained summary-level data on VTE and its subtypes (PE and DVT) from the FinnGen study (release 6, https://r6.finngen.fi/), which encompass 11,288 VTE cases, 5,130 PE cases, 5,632 DVT cases, and up to 254,771 controls, and all participants were of European descent. Association tests in FinnGen had been adjusted for age, sex, first 10 genetic principal components, and the genotyping batch.

The details of the data source and IVs are all shown in Table 1; Supplementary Table S1.

**TABLE 1:** Details of GWAS included in Mendelian randomization analyses. BMI, body mass index; PAI-1, plasminogen activator inhibitor-1; MCP-1, monocyte chemoattractant protein-1; RETN, resistin; GIANT, Genetic Investigation of ANthropometric Traits; SCALLOP, Systematic and Combined AnaLysis of Olink proteins; VTE, venous thromboembolism; DVT, deep vein thrombosis; PE, pulmonary embolism.

Phenotype	Sample size	Ethnicity	Consortium/author	ID
Exposure				
BMI	339,224	European	GIANT	ieu-a-2
Adiponectin	29,347	European	Dastani Z	—
Leptin	49,909	Multi-ancestry	Yaghootkar H	ebi-a-GCST90007310
PAI-1	34,448	European	McCartney D L	—
MCP-1	21,758	European	SCALLOP	—
Leptin receptor	3,301	European	Sun Benjamin B	prot-a-1724
RETN	21,758	European	SCALLOP	—
Outcomes	Cases/Controls			
VTE	11,288/254,771	European	FinnGen	R6_I9_VTE
DVT	5,632/254,771	European	FinnGen	R6_I9_ PHLETHROMBDVTLOW
PE	5,130/254,771	European	FinnGen	R6_I9_PULMEMB

### Statistical analysis

#### Data preparation

By matching genetic IVs, we first harmonized GWAS summary-level data, and used allele frequencies to identify and correct the palindromic variants. SNPs with major allele frequencies greater than 45% were excluded as it was impossible to estimate the strand orientation with sufficient accuracy. F-statistic and *R* ^2^ were used to evaluate the effectiveness of IVs. *R* ^2^ is the proportion of variance of BMI and all mediators explained by the IV, which can be calculated using 2 × *β* ^2^ × EAF×(1-EAF), where *β* is the estimated coefficient of the IV in BMI and all mediators like GWAS, and EAF is the effect allele frequency. The F-statistic can be calculated by the following formula: F = *β*_exposure_^2^/SE_exposure_^2^ (Li and Martin, 2002). If the F-statistic is less than 10, there may be bias, owing to weak instrumental variable effects (Sanderson et al., 2021).

### Mendelian randomization

The basic assumptions of MR are as follows (Burgess and Thompson, 2015):

1. The genetic instrumental variable(s) are robustly related to the risk factor of interest.
2. There is no relationship between any confounders of the risk factor and outcome and the genetic instrumental variable.
3. There is no path from the genetic instrument to the outcome other than through its relationship with the risk factor.

Previous research studies indicated that the third hypothesis, which states that there is horizontal pleiotropy of IVs, is the most likely to be broken and provide biased results. When genetic variation influences outcomes by affecting other factors independent of exposure, this phenomenon is referred to as horizontal pleiotropy of genetic instrumental variables, which may potentially mislead the causal estimates (Xu et al., 2017). To avoid bias from horizontal pleiotropy, MR-Egger regression was used to detect the horizontal pleiotropy. If the MR-Egger intercept *p*-value < 0.05, we deem these SNPs have horizontal pleiotropy; the MR-PRESSO (Mendelian Randomization Pleiotropy RESidual Sum and Outlier) method was used to detect and correct the potential outliers (Verbanck et al., 2018). Additionally, we used I2-GX calculated from MR-Egger regression and Cochran’s Q test to assess the expected relative bias (or dilution) due to the measurement error and between-SNP heterogeneity, respectively (Bowden et al., 2016). A value of I2-GX close to 100% indicates that dilution does not materially affect the standard MR-Egger analyses for these data. If Cochran’s Q test *p*-value was < 0.1, we deem these SNPs have between-SNP heterogeneity.

The inverse-variance weighted (IVW) method is used as the main method to assess the causal association between exposures and outcomes; if between-SNP heterogeneity is present, we used the IVW method with the fixed-effects model; if not, we used IVW with the multiplicative random effects model. MR-Egger, weighted median, and weighted mode methods were used in sensitivity analyses to evaluate the robustness of the MR results. Finally, the leave-one-out analysis was conducted to detect the influential SNPs. To account for multiple comparisons (seven exposures and three outcomes), a Benjamini–Hochberg (FDR) correction was employed. *p*-values less than 0.05, but q-values more than 0.05, indicated a plausible causal relationship.

### Mediation analysis

**FIGURE 1:**
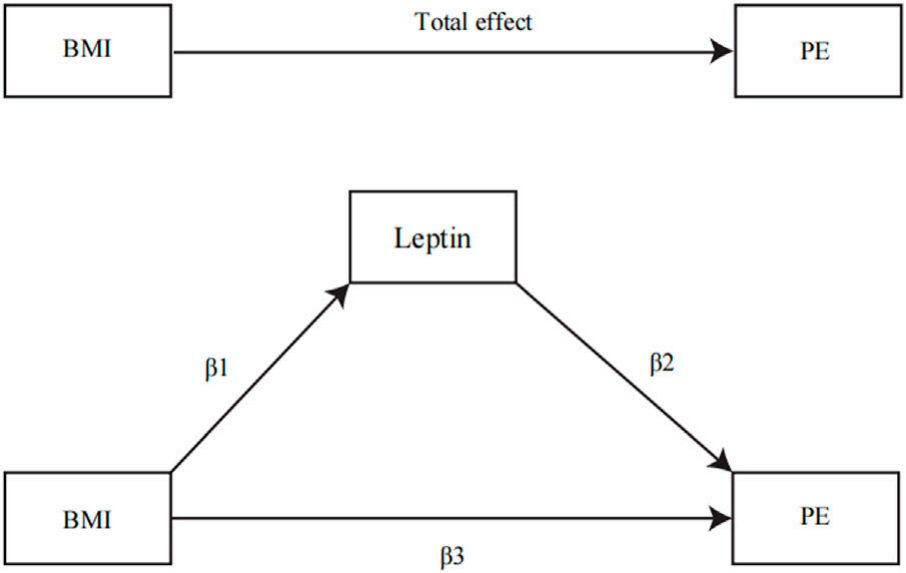
Illustration of parameters estimated to obtain total, direct, and indirect effects: β1, the MR effects of BMI on the potential mediator; β2, the MR effect of the circulating leptin levels with PE adjusted for genetically determined BMI; β3, the MR effect of BMI on PE adjusted for the genetically determined circulating leptin levels. The total effect was determined by β1*β2 + *β*3.

IVW was used as the main method to calculate the influence of BMI on the potential mediator. The influence of the potential mediator on VTE, DVT, and PE risks was estimated by using regression based on MVMR while accounting for the genetic impact of instruments on BMI (Burgess et al., 2015). In addition, we calculated the indirect effect of the mediator on outcomes using the product of coefficient method and estimated the proportion mediated (Sanderson, 2021) (Figure 1), where the regression coefficient β1 is the MR effect of BMI on the potential mediator, β2 is the MR effect of the potential mediator *k* with outcomes adjusted for genetically determined BMI, and β3 is the MR effect of BMI on outcomes adjusted for the genetically determined potential mediator (Xu et al., 2017). In addition, the indirect effect was β1*β2, while the direct effect was β3. All regression coefficients were derived from MR instrumental analysis using IVW. In the end, Sobel’s test was applied to test the significance of the mediation effects (Chong et al., 2022). The following equation was used to estimate the percentage of the impact that is mediated by any of the potential mediators (Xu et al., 2017).

$$E\left(\%\right)=\sum_{K=1}^{K}\beta 1*\beta 2k/\sum_{K=1}^{K}\beta 3+\beta 1*\beta 2k.$$

All MR analyses were conducted using R (version 4.2.1) and the TwoSampleMR R package version 0.5.6.

## Results

### Statistics for the effectiveness of IVs

For MR analysis, the number of included SNPs ranged from 3 to 84, explaining 0.0038%–17% of the total variance in exposures (Table 2), and the F-statistic ranged from 29.91 to 1,597.86 (Supplementary Table S1). All F-statistic values were >10, typically recommended for MR analyses. The I2-GX value ranged from 98.41% to 99.52% (Table 2).

**TABLE 2:** Heterogeneity and pleiotropy analysis.

Outcome	Exposures	Efficiency of SNPs			Between-SNP heterogeneity		Horizontal pleiotropy	
		Number of SNPs	R2	I2-GX (%)	Q statistics	*p*-value	Egger intercept	*p*-value
VTE	Adiponectin	12	0.0120	99.03	22.47	0.02	0.008234203	0.46
	Leptin	4	0.0021	98.52	2.55	0.47	−0.028665233	0.68
	PAI-1	4	0.0007	99.20	2.50	0.48	0.068544866	0.41
	MCP-1	6	0.0076	98.60	10.52	0.06	−0.007249059	0.82
	Leptin receptor	3	0.1744	99.52	0.29	0.86	−0.012825091	0.69
	RETN	12	0.0137	98.43	16.28	0.13	−0.00684842	0.61
	BMI	83	0.0064	98.41	148.75	0.00	−0.006694425	0.26
DVT	Adiponectin	12	0.0120	99.03	26.93	0.01	0.013735576	0.41
	Leptin	4	0.0021	98.52	2.95	0.40	−0.031977555	0.76
	PAI-1	4	0.0007	99.20	3.37	0.34	−0.018223137	0.89
	MCP-1	6	0.0076	98.60	8.07	0.15	−0.016683525	0.66
	Leptin receptor	3	0.1744	99.52	1.67	0.43	0.034036455	0.49
	RETN	12	0.0137	98.43	19.41	0.05	−0.002252887	0.91
	BMI	83	0.0064	98.41	145.89	0.00	−0.009078654	0.27
PE	Adiponectin	12	0.0120	99.03	17.62	0.09	0.00713946	0.61
	Leptin	4	0.0021	98.52	3.78	0.29	−0.018005193	0.88
	PAI-1	4	0.0007	99.20	0.54	0.91	0.058116394	0.60
	MCP-1	6	0.0076	98.60	9.08	0.11	−0.032888159	0.41
	Leptin receptor	3	0.1744	99.52	6.04	0.05	−0.08423213	0.25
	RETN	12	0.0137	98.43	20.74	0.04	−0.001965404	0.93
	BMI	83	0.0064	98.41	119.62	0.01	−0.010056853	0.19
Leptin	BMI	33	0.0038	98.73	63.46	0.00	0.001330344	0.76

### MR analysis

#### Causal effect of adipokines on outcomes

**FIGURE 2:**
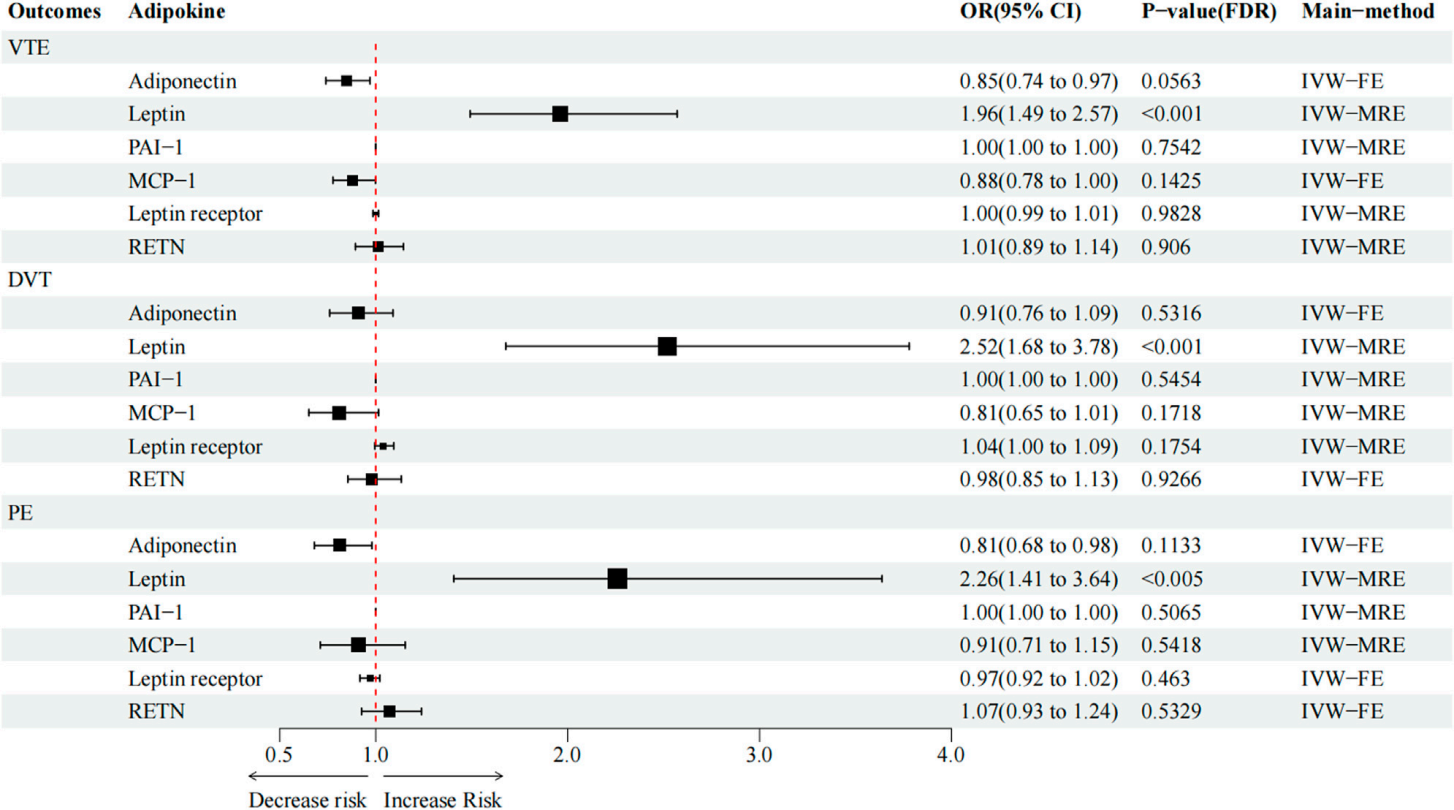
Causal effect of circulating adipokine concentrations on and VTE, DVT, and PE. OR, odds ratio; CI, confidence interval; IVW, inverse variance weighted; RE, random-effects; FE, fixed-effects.

High circulating leptin levels were associated with higher odds of VTE (OR = 1.96; *p* < 0.001; q < 0.001), DVT (OR = 2.52; *p* < 0.001; q < 0.001), and PE (OR = 2.26; *p* < 0.001; q = 0.005) (Figure 2). Notably, the causal effect between elevated serum adiponectin and the decreased risk of VTE (OR = 0.85; *p* = 0.013; q = 0.053) and PE (OR = 0.81; *p* = 0.032; q = 0.083), and between MCP-1 and the decreased risk of VTE (OR = 0.88; *p* = 0.048; q = 0.143) was no longer significant after FDR adjustment (Table 3; Figure 2). For the other three types of adipokines, no statistically significant association with VTE, DVT, and PE was detected. To evaluate the IVW approaches’ robustness, we performed sensitivity analyses using the other MR methods (MR-Egger, weighted median, and weighted mode).

**TABLE 3:** Main result and sensitivity analysis of Mendelian randomization in circulating adipokine concentrations and VTE, DVT, and PE.

Outcome	Exposures	IVW-FE			IVW-MRE			MR-Egger			Weighted median			Weighted mode		
		OR	95% CI	*p*-value	OR	95% CI	*p*-value	OR	95% CI	*p-*value	OR	95% CI	*p*-value	OR	95% CI	*p*-value
VTE	Adiponectin	0.85	0.74–0.97	0.01	0.85	0.70–1.02	0.08	0.92	0.70–1.21	0.56	0.88	0.75–1.04	0.15	0.89	0.76–1.03	0.15
	Leptin	1.96	1.46–2.63	0.00	1.96	1.49–2.57	0.00	3.51	0.32–38.46	0.41	2.00	1.39–2.87	0.00	2.21	1.28–3.83	0.07
	PAI-1	1.00	0.99–1.01	0.66	1.00	0.99–1.01	0.63	1.00	0.99–1.01	0.54	1.00	0.99–1.01	0.68	1.00	0.99–1.01	0.76
	MCP-1	0.88	0.78–1.00	0.05	0.88	0.73–1.06	0.17	0.93	0.58–1.5	0.78	0.90	0.76–1.06	0.21	0.87	0.69–1.1	0.30
	Leptin receptor	1.00	0.96–1.04	0.99	1.00	0.99–1.01	0.98	1.02	0.95–1.09	0.73	1.00	0.96–1.04	0.96	1.00	0.96–1.05	0.84
	RETN	1.01	0.91–1.12	0.82	1.01	0.89–1.14	0.86	1.07	0.83–1.39	0.60	1.03	0.89–1.19	0.66	1.04	0.90–1.21	0.58
DVT	Adiponectin	0.91	0.76–1.09	0.32	0.91	0.69–1.21	0.53	1.04	0.69–1.59	0.84	0.98	0.79–1.22	0.86	0.98	0.80–1.21	0.88
	Leptin	2.52	1.67–3.79	0.00	2.52	1.68–3.78	0.00	4.83	0.12–187.96	0.49	2.63	1.59–4.37	0.00	3.18	1.46–6.92	0.06
	PAI-1	1.00	0.99–1.01	0.27	1.00	0.99–1.01	0.30	1.00	0.99–1.01	0.66	1.00	0.99–1.01	0.16	1.00	0.99–1.01	0.25
	MCP-1	0.81	0.68–0.97	0.02	0.81	0.65–1.01	0.07	0.92	0.52–1.63	0.79	0.78	0.62–0.99	0.04	0.78	0.59–1.04	0.15
	Leptin receptor	1.04	0.99–1.1	0.11	1.04	1.00–1.09	0.08	1.00	0.90–1.10	0.96	1.04	0.99–1.1	0.15	1.03	0.98–1.09	0.34
	RETN	0.98	0.85–1.13	0.82	0.98	0.82–1.19	0.87	1.00	0.68–1.49	0.96	0.94	0.77–1.14	0.51	0.91	0.72–1.17	0.49
PE	Adiponectin	0.81	0.68–0.98	0.03	0.81	0.34–1.03	0.0892	0.87	0.61–1.25	0.48	0.86	0.67–1.09	0.21	0.86	0.69–1.05	0.17
	Leptin	2.26	1.48–3.45	0.00	2.26	1.41–3.64	0.0008	3.26	0.04–261.61	0.65	2.06	1.19–3.57	0.01	1.68	0.67–4.2	0.35
	PAI-1	1.00	0.99–1.01	0.70	1.00	0.99–1.01	0.3658	1.00	0.99–1.01	0.74	1.00	0.99–1.01	0.51	1.00	0.99–1.01	0.55
	MCP-1	0.91	0.76–1.08	0.28	0.91	0.71–1.15	0.4214	1.16	0.65–2.07	0.65	0.93	0.74–1.16	0.51	0.97	0.75–1.26	0.84
	Leptin receptor	0.97	0.92–1.02	0.23	0.97	0.88–1.06	0.4911	1.08	0.98–1.2	0.37	0.97	0.92–1.03	0.33	1.00	0.94–1.06	1.00
	RETN	1.07	0.93–1.24	0.36	1.07	0.88–1.31	0.5008	1.09	0.72–1.66	0.70	1.01	0.82–1.24	0.95	1.08	0.85–1.37	0.53

OR, odds ratio; CI, confidence interval; IVW, inverse variance weighted; RE, random-effects; FE, fixed-effects.

As shown in Table 3; Supplementary Figure S2, in terms of the causal relationship between circulating leptin levels and VTE, DVT, and PE, sensitivity studies yielded comparable findings. The IVW and the weighted median methods discovered a significant association between circulating leptin levels and the risk of VTE, DVT, and PE, and all four MR methods suggest that circulating leptin levels were a risk factor for VTE, DVT, and PE (OR:1.96–3.51). No significant association with the other three adipokines was relevant to the risk of VTE, DVT, and PE, using any of the methods. The leave-one-out analysis revealed, even after eliminating any SNPs, that the correlation between circulating leptin levels and VTE, DVT, and PE (Supplementary Figure S9) remained significant. A significant relationship between other adipokines and outcomes was not identified.

#### Causal effect of BMI on circulating leptin levels and outcomes

It was discovered that a high BMI value was closely linked with higher odds of VTE (OR = 1.45, *p* < 0.001), DVT (OR = 1.63, *p* < 0.001), and PE (OR = 1.37, *p* < 0.001). High BMI was found to be associated with elevated circulating leptin levels (beta = 0.61; *p* < 0.001; IVW-FE) (Table 4). Consistent results were achieved in a sensitivity analysis using various MR techniques such as the IVW-FE method (Table 4, Supplementary Figure S7).

**TABLE 4:** Main result and sensitivity analysis of Mendelian randomization in BMI and VTE, DVT, PE, and leptin.

Outcome	Method	OR	95% CI	*p*-value
VTE	IVW-FE	1.45	1.28–1.65	1.55E-08
	IVW-MRE	1.45	1.22–1.73	2.39E-05
	MR-Egger	1.82	1.19–2.77	7.12E-03
	Weighted median	1.61	1.29–2.00	1.77E-05
	Weighted mode	1.58	1.19–2.10	2.15E-03
DVT	IVW-FE	1.63	1.36–1.95	8.94E-08
	IVW-MRE	1.63	1.29–2.07	5.50E-05
	MR-Egger	2.21	1.23–3.96	9.21E-03
	Weighted median	2.21	1.64–2.97	1.50E-07
	Weighted mode	2.35	1.65–3.35	1.02E-05
PE	IVW-FE	1.37	1.14–1.65	8.82E-04
	IVW-MRE	1.37	1.10–1.71	5.60E-03
	MR-Egger	1.92	1.11–3.30	2.13E-02
	Weighted median	1.61	1.18–2.19	2.68E-03
	Weighted mode	1.83	1.23–2.74	4.13E-03
Leptin	Method	Beta	SE	*p*-value
	IVW-FE	0.61	0.0411	1.93E-50
	IVW-MRE	0.61	0.0578	2.79E-26
	MR-Egger	0.58	0.1282	8.60E-05
	Weighted median	0.64	0.0647	9.78E-23
	Weighted mode	0.64	0.0699	1.61E-10

### Mediation analysis

**FIGURE 3:**
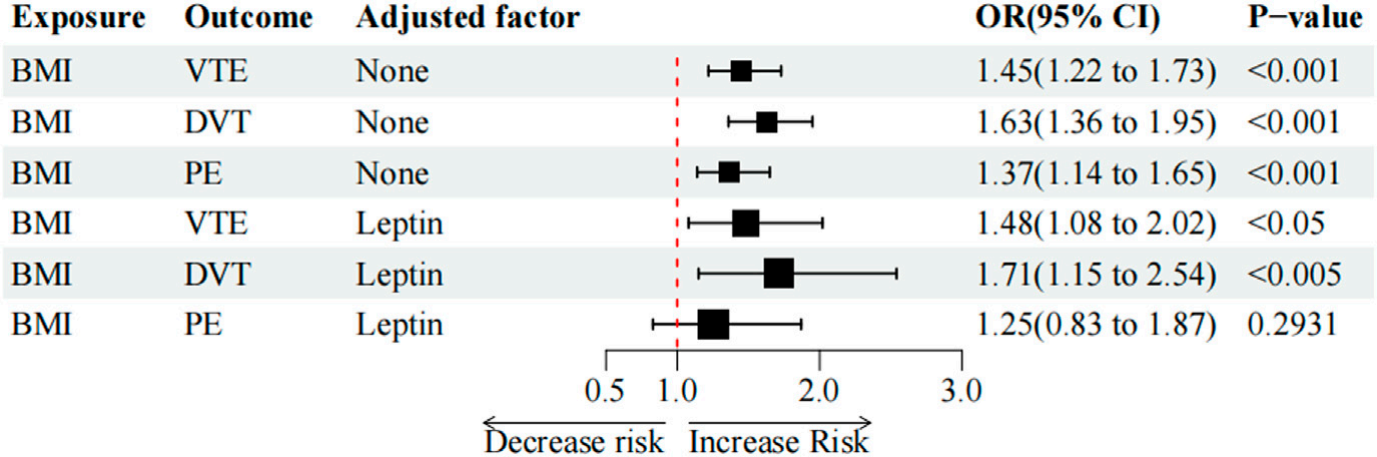
Multivariate MR analysis of BMI on VTE, DVT, and PE.

As the two-sample MR analysis revealed only the causality link between circulating leptin levels and VTE and its subtypes, only the mediating function of serum leptin levels in BMI on VTE and the risk of its subtypes was further investigated. In the multivariable IVW analysis, after controlling for circulating leptin levels, the causal impact of BMI on PE (OR = 1.25, *p* = 2.93e-01) was no longer significant and reduction in significance was observed for the effect on VTE (OR = 1.48, *p* = 1.22e-02) and DVT (OR = 1.71, *p* = 7.85e-03) (Table 5; Figure 3). The great difference between the univariable and multivariable MR results suggested that the causal influence of BMI on VTE, DVT, and PE could be mediated by circulating leptin levels. The mediation effect of circulating leptin levels in the causal pathway from BMI to PE was 1.28 (95% CI, 0.95, 1.71; *p* = 0.10), accounting for 39.14% of the total effect (Table 6).

**TABLE 5:** Multivariate MR analysis.

Exposure	Outcome	Adjusted factor	Multivariate MR analysis		
			OR	95% CI	*p*-value
BMI	VTE	None	1.45	1.22–1.73	1.55E-08
BMI	DVT	None	1.63	1.36–1.95	8.94E-08
BMI	PE	None	1.37	1.14–1.65	8.82E-04
BMI	VTE	Leptin	1.48	1.08–2.02	1.22E-02
BMI	DVT	Leptin	1.71	1.15–2.54	7.85E-03
BMI	PE	Leptin	1.25	0.83–1.87	2.93E-01
Leptin	VTE	BMI	1.26	0.88–1.80	2.01E-01
Leptin	DVT	BMI	1.32	0.84–2.01	2.34E-01
Leptin	PE	BMI	1.49	0.92–2.39	1.02E-01

**TABLE 6:** Mediation analysis.

	Direct effect (OR, 95%CI)	Indirect effect (OR, 95%CI)	Proportion mediated (%)	z	*p*-value
BMI	1.25 (0.83–1.87)	1.28 (0.95–1.71)	39.14	1.62	0.10

BMI, body mass index; OR, odds ratio; CI, confidence interval; z, statistic of Sobel’s test.

## Discussion

In this large-scale MR analysis, we found a causal relationship between genetically determined circulating leptin levels and the risk of VTE, DVT, and PE. In addition, it was acknowledged that adiponectin may be a protective factor for VTE and PE. Simultaneously, MCP-1 may be a protective factor for VTE. However, we found no evidence supporting the causal associations between genetically determined levels of the other three adipokines (RETN, leptin receptor, and PAI-1) and the risk of VTE, DVT, and PE. These findings implied that adiponectin, PAI-1, MCP-1, leptin receptor, and RETN might have no significance in the pathogenesis of VTE, DVT, and PE. Although the difference between univariate Mendelian randomization and multivariate Mendelian randomization results suggests that leptin may mediate the effect of BMI on PE, the results of the mediation analysis suggest that this mediating effect of leptin did not reach statistical differences (*p* > 0.05).

Leptin is a 16-kDa hormone released by adipocytes, the secretion of which is dependent on the fat depot. Additionally, leptin production is greater in the subcutaneous fat depot than that in the visceral depot. The blood levels of leptin show a proportional distribution pattern in adipose tissue mass, which also manifests the instantaneous changes in nutritional status, as reflected by a sharp decrease after the onset of fasting (Raucci et al., 2013). Previous observational studies on the relationship between circulating leptin levels and VTE risk have shown inconclusive findings to some extent. Observational studies and *in vitro* tests have demonstrated that the circulating leptin levels are related to an extensive array of hemostatic factors, including platelet activation parameters, procoagulant factors, and fibrinolytic factors. Leptin has been demonstrated to stimulate TF activity, antigen and mRNA expression, and the production of microparticles containing TF in human peripheral mononuclear cells (Napoleone et al., 2007; Rafail et al., 2008; Petrini et al., 2016). It is worth mentioning that Ayer et al. found that the elevation of the tissue factor (TF) activity in mononuclear cells was only accomplished at supra-physiological quantities of leptin but not at the lower amounts often encountered in obese individuals (Ayer et al., 2010). A prospective study of 264 patients with acute pulmonary embolism revealed that a low plasma leptin concentration was an independent risk factor for high mortality among patients with acute pulmonary embolism (Dellas et al., 2013). A prospective study of 203 OA patients reveals that a high preoperative leptin level may be an independent risk factor for postoperative DVT (OR = 2.17, 95% CI, 1.01–4.64) (Lu et al., 2018). However, a recent nested case–control analysis (416 VTE cases and 848 controls) based on the trauma cohort revealed that blood leptin levels were related to the incidence of VTE, before adjusting for BMI, which indicates that the association between circulating leptin levels and VTE risk might be confounded by BMI (Frischmuth et al., 2022). This is consistent with the results of the current MR study. In the mediation analysis, the mediating effect of circulating leptin levels failed Sobel’s test (*p* = 0.10), suggesting leptin may not be a strong mediator of BMI on the risk of PE.

Adiponectin is primarily released by the visceral adipose tissue and is the most abundant adipokine, with quantities in the bloodstream inversely associated with obesity, which specifically exerts anti-apoptotic, anti-inflammatory/anti-fibrotic, and insulin-sensitizing effects (Straub and Scherer, 2019). In a rat model of hyperlipidemia, adiponectin inhibits platelet aggregation by enhancing eNOS activation and reducing oxidative/nitrosative stress, such as by inhibiting iNOS expression and superoxide production (Wang et al., 2011). In an observational study, Restituto et al. (2010) found that adiponectin decreased platelet aggregation and activation and demonstrated antithrombotic characteristics in metabolic syndrome patients. A retrospective study involving 95 patients with pulmonary embolism revealed that patients with pulmonary embolism had lower adiponectin levels than normal controls (Gul et al., 2016). Recent prospective cohort research also demonstrated that low adiponectin levels are associated with post-thrombotic syndrome, regardless of BMI (Mrozinska et al., 2018). In the current MR study, we found that adiponectin was associated with a decreased risk of VTE (OR = 0.85; *p* = 0.013; q = 0.053) and PE (OR = 0.81; *p* = 0.032; q = 0.083). Although the FDR-corrected significance threshold was not met, adiponectin could be considered a potential protective factor for VTE and PE when combined with previous findings.

MCP-1 is from the family of CC chemokines, which can bind to several receptors but mainly enforces its biological effect by attaching to the extracellular region of CCL2 (chemokine ligand 2). The primary sources of monocyte chemoattractant protein-1 are epithelial cells, endothelial cells, smooth muscle cells, monocytes/macrophages, fibroblasts, astrocytes, and microglial cells (Singh et al., 2021). Obesity-related insulin resistance promotes the inflammation of the adipose tissue through MCP-1 from adipocytes, resulting in the recruitment of monocytes and activation of macrophages (Shimobayashi et al., 2018). As reported, monocyte recruitment plays a pivotal role in thrombus lysis; endogenous MCP-1 levels increased in spontaneously dissolving venous thrombi, and the injection of MCP-1 into the thrombosed tissue enhances thrombus lysis, according to previous research studies (Humphries et al., 1999; Ali et al., 2006; Gutmann et al., 2020). Zhang et al. reported the subsequent elevation experiment on rabbits, where they discovered that increased MCP-1 protein expression caused by bone marrow MSC transplantation promoted the regression and recanalization of DVT (Zhang et al., 2019). Georgakis et al. discovered that higher MCP-1 levels were related to an increased risk of cardioembolic arterial thrombosis in stroke, as well as mortality (Georgakis et al., 2019a; Georgakis et al., 2019b; Georgakis et al., 2021). MCP-1 was associated with a decreased risk of VTE in our study (OR = 0.88, *p* = 0.048). However, this association was no longer significant after FDR correction (q = 0.14), suggesting the association observed in the initial analysis may have been false positive, and further research would be needed to determine if there is a true association between MCP-1 and VTE. Although there are some distinctions in the processes of formation and pathological alterations for arterial and venous thrombosis, there are many similarities in the pathogenesis (Lijfering et al., 2011; Prandoni, 2020). Given that the conclusion regarding the positive contribution of MCP-1 in the process of venous thrombus dissolution is derived from *in vitro* experiments and the association of MCP-1 with an elevated risk of cardiovascular embolic events is supported by higher quality studies, further research with robust evidence is warranted to establish the precise role played by MCP-1 in venous thromboembolism.

Here, we found no association between the genetically determined PAI-1 concentration and the risk of VTE, DVT, and PE. Zhang et al. (2020) and Wang et al. (2022) revealed the PAI-1 4G/5G polymorphism associated with the risk of the onset of VTE in East Asian ancestry, but the causal relationship between serum PAI-1 levels and VTE risk was not recognized in our study, probably because the GWAS data we assessed were generated from European ancestry. Because of variances in gene linkage disequilibrium among races, SNPs indicating PAI-1 levels in GWAS studies of European populations do not reflect PAI-1 levels in other races.

Several aspects of our research study make it superior to others. 1) Statistical data summary of exposure and outcomes was from the largest and latest GWAS in related fields, and there was no overlapping sample in our study. 2) Strict criteria were established to select IVs which may strengthen the statistical power. 3) Since genetic variants were scattered over multiple chromosomes, it is possible that potential gene–gene interactions will not have much effect on the results. 4) To enhance the accuracy of the estimation, we utilized different methods for sensitivity analysis. 5) A mediation analysis based on MVMR was used to investigate the mediating role of adipokines in the risk of obesity and VTE. This study does have some limitations. 1) GWAS used included only the European ancestry population. Thus, additional studies should be performed to explore the intermediated effect in the non-European population. 2) There were some heterogeneities in the analysis. Because meta-GWAS data were used, it was impossible to investigate the possibility of non-linear connections or stratification effects that varied according to age, health status, or gender. 3) Since the GWAS research employed as an endpoint was not stratified by gender, it could not elucidate if adipokines’ influence on VTE was subjected to the gender differences. 4) Limited adipokines were included in the current study. 5) Further research studies were required to determine if the adipokines that showed negative results in current investigation can trigger the formation of DVT, VTE, or PE on their own or contribute to the pathogenesis of DVT, VTE, or PE in collaboration with other adipokines. 6)

## Conclusion

Our MR investigation revealed that the circulating leptin level was a risk factor for VTE, DVT, and PE, but it may not be a mediator of BMI on the risk of VTE, DVT, and PE and that adiponectin was a potential protective factor for both VTE and PE.

## Data Availability

The datasets presented in this study can be found in online repositories. The names of the repository/repositories and accession number(s) can be found in the article/Supplementary Material.
