# Supplementary figures and images for: Circulating adipokine concentrations and the risk of venous thromboembolism: A Mendelian randomization and mediation analysis
Source: Front Genet. 2023 Mar 28;14:1113111. doi: 10.3389/fgene.2023.1113111 (PMC10086141; doi:10.3389/fgene.2023.1113111)

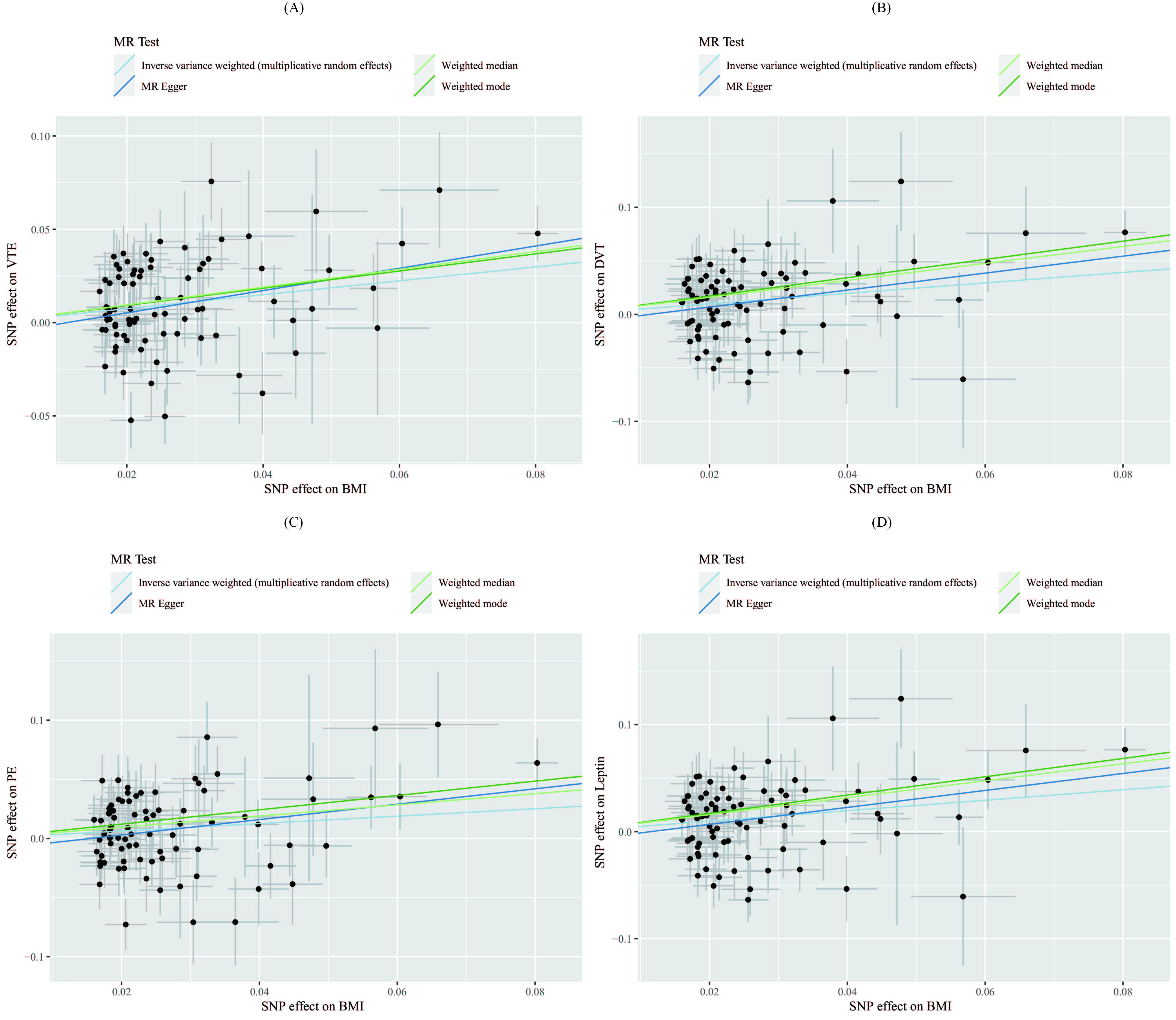

Supplement: Supplementary file 1 [file Image15.JPEG]

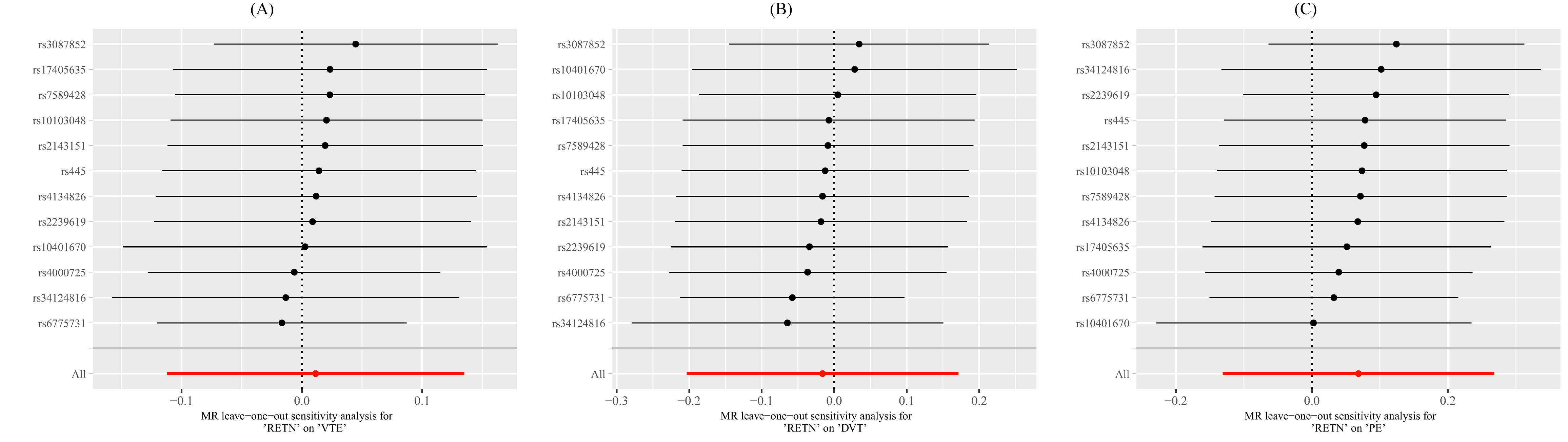

Supplement: Supplementary file 2 [file Image6.TIF]

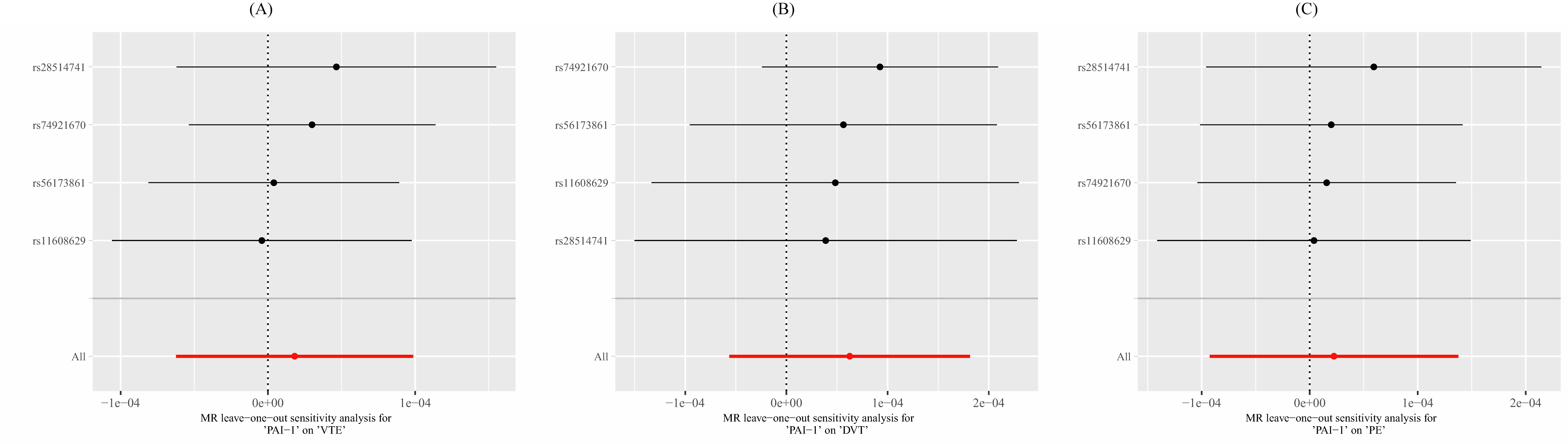

Supplement: Supplementary file 3 [file Image3.TIF]

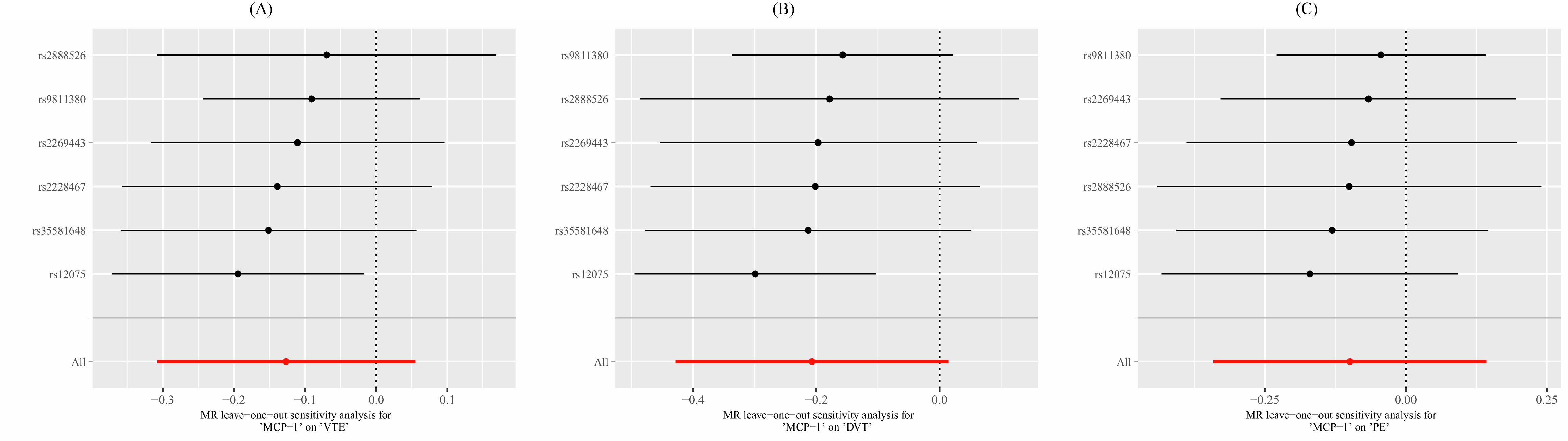

Supplement: Supplementary file 4 [file Image4.TIF]

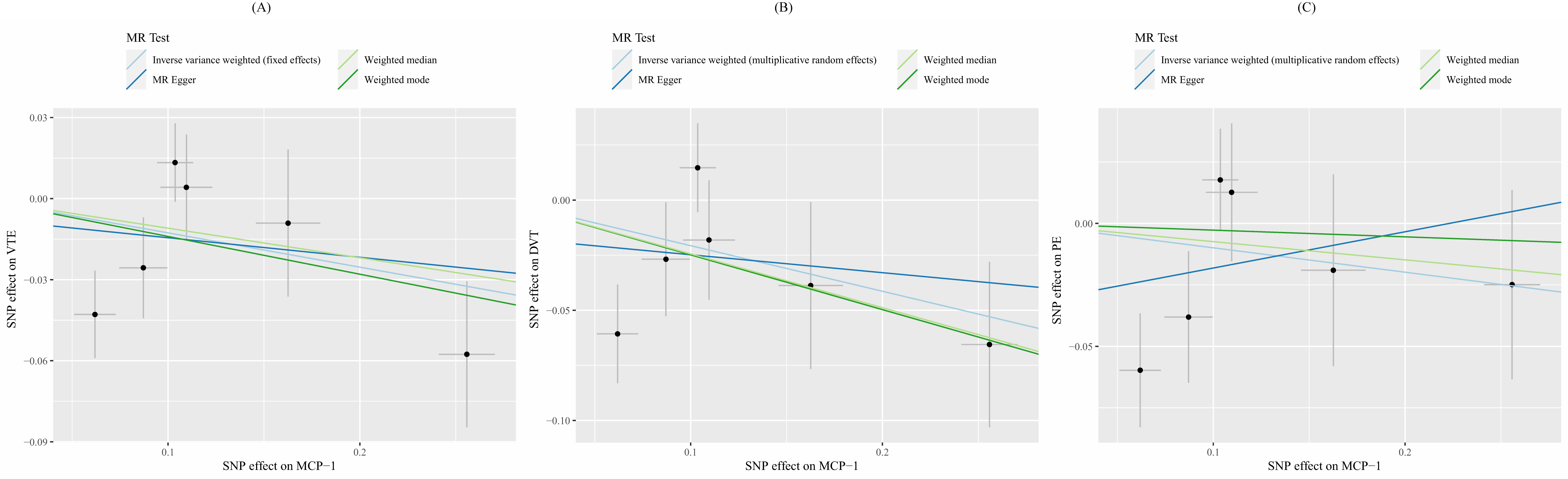

Supplement: Supplementary file 5 [file Image9.TIF]

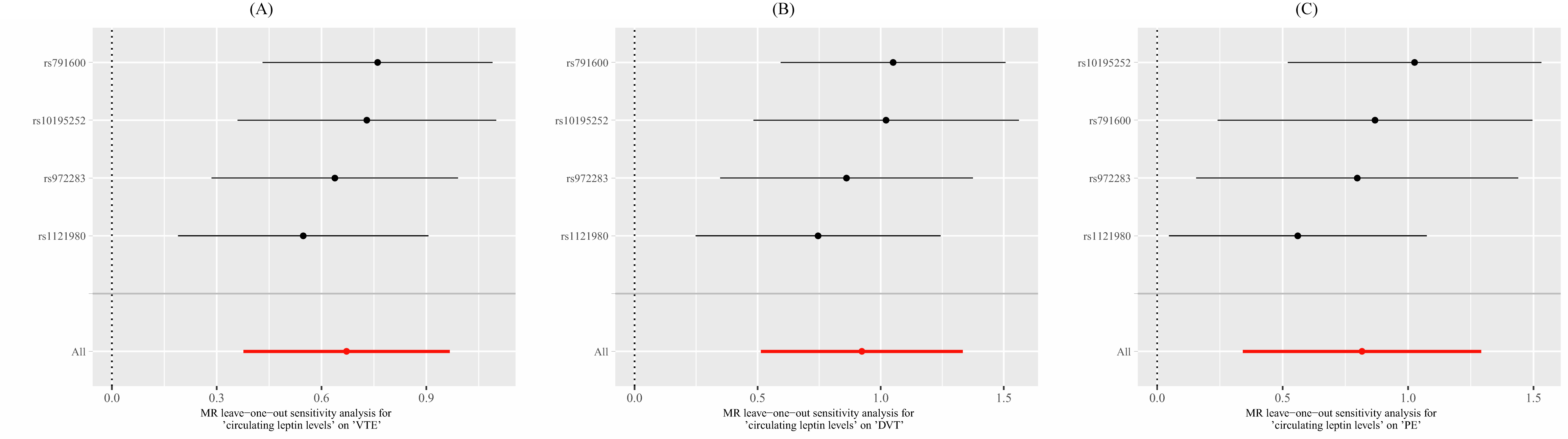

Supplement: Supplementary file 6 [file Image2.TIF]

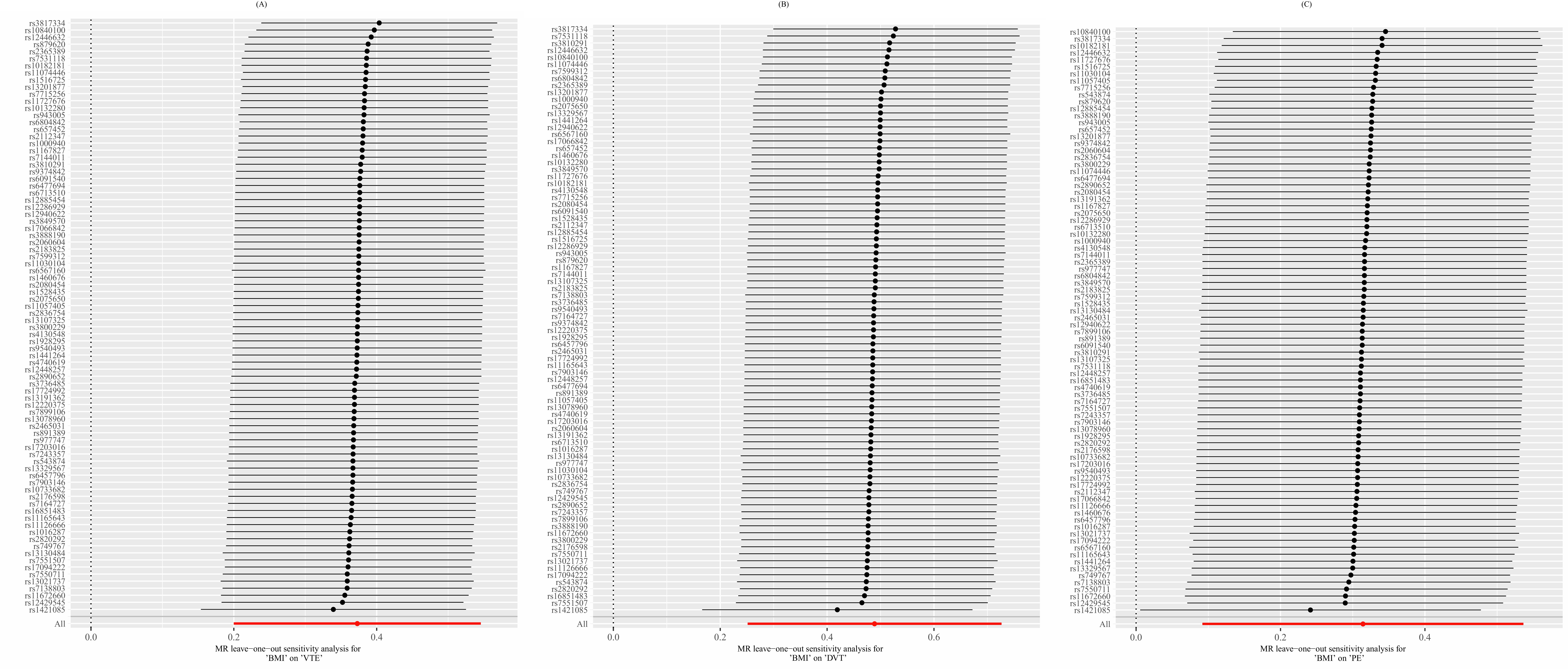

Supplement: Supplementary file 7 [file Image10.JPEG]

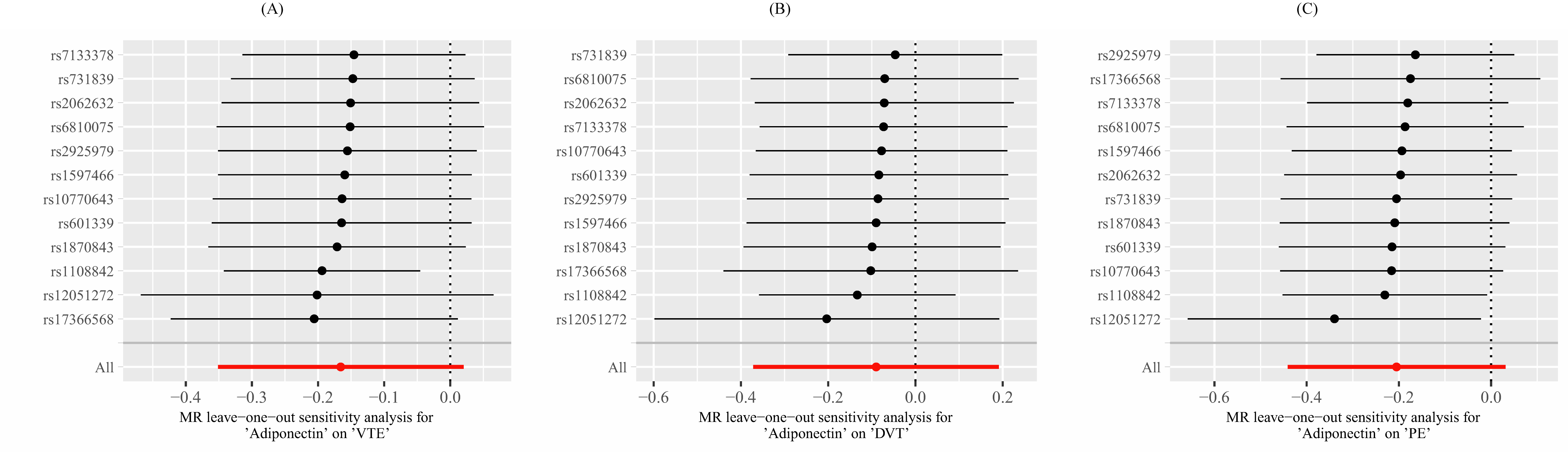

Supplement: Supplementary file 8 [file Image1.TIF]

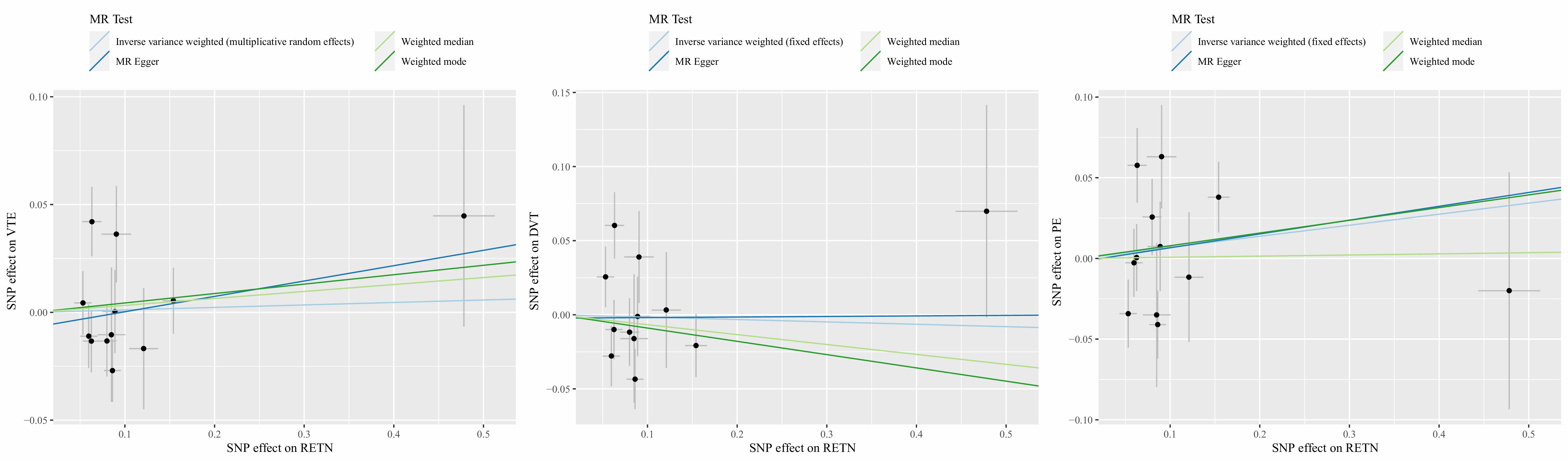

Supplement: Supplementary file 9 [file Image14.JPEG]

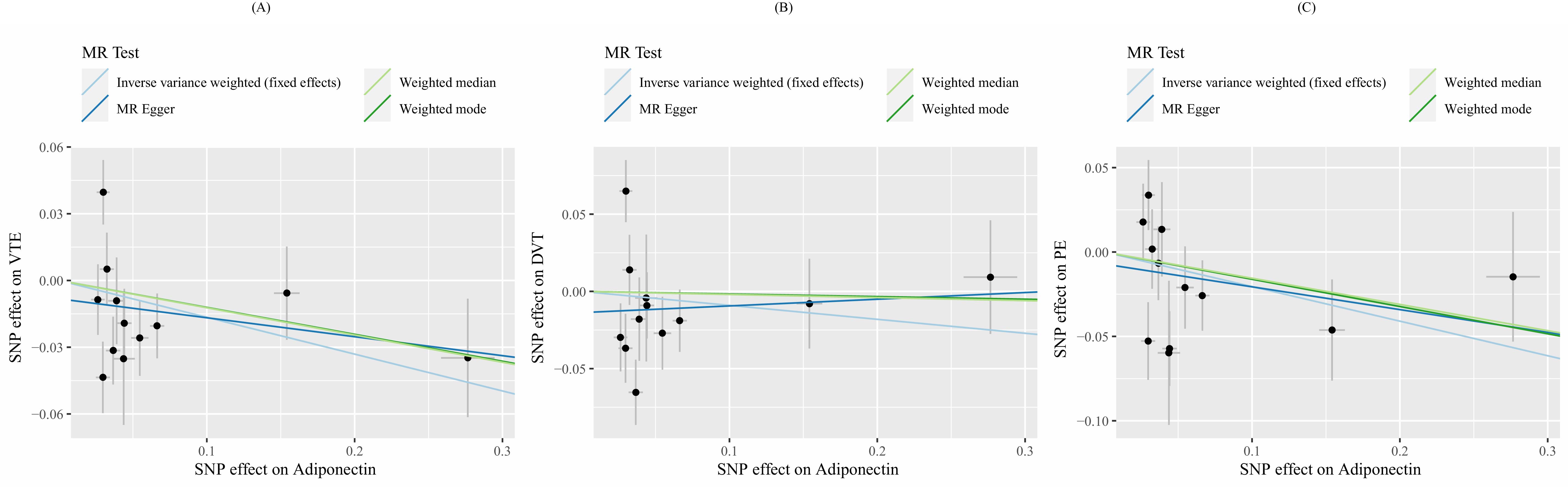

Supplement: Supplementary file 10 [file Image12.JPEG]

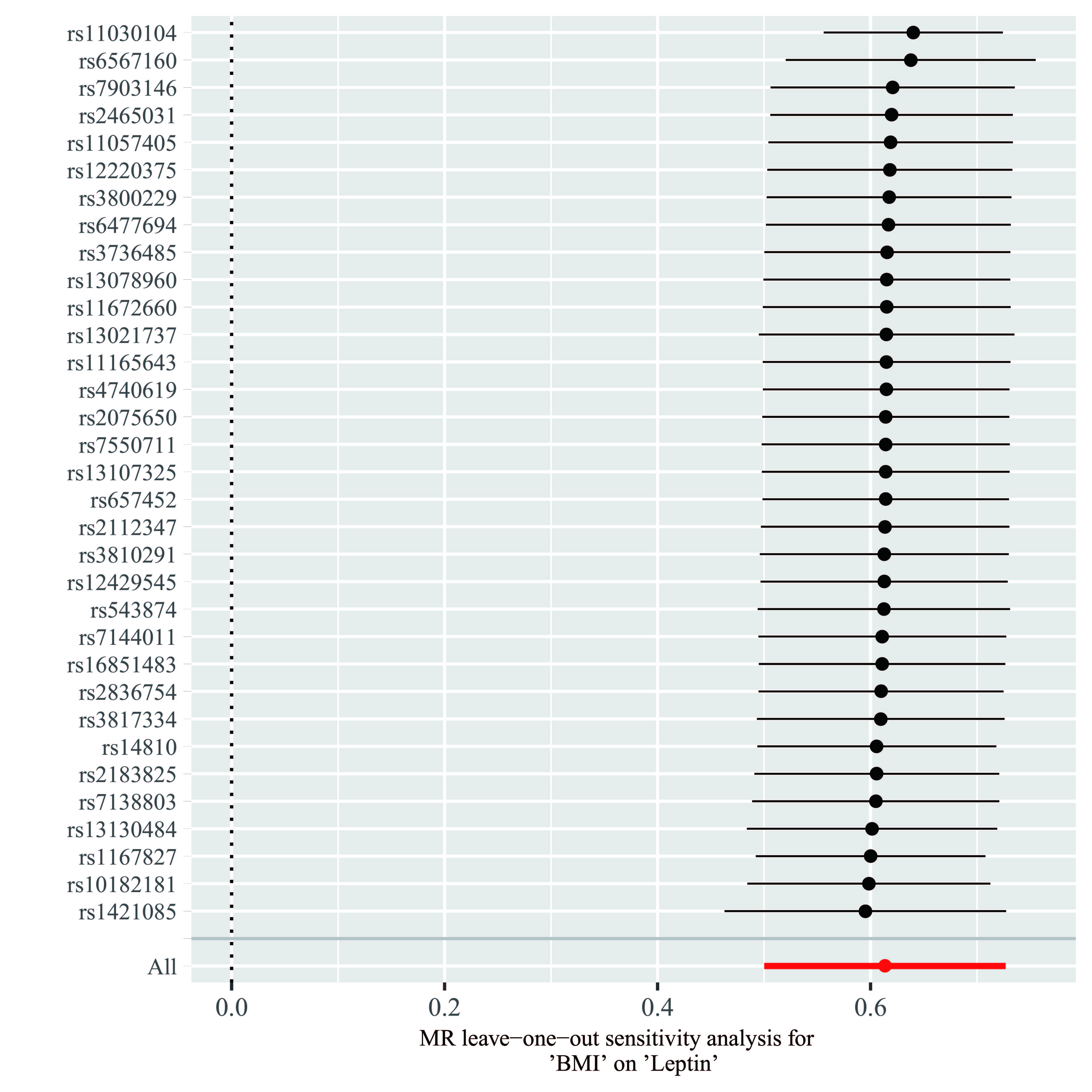

Supplement: Supplementary file 11 [file Image11.JPEG]

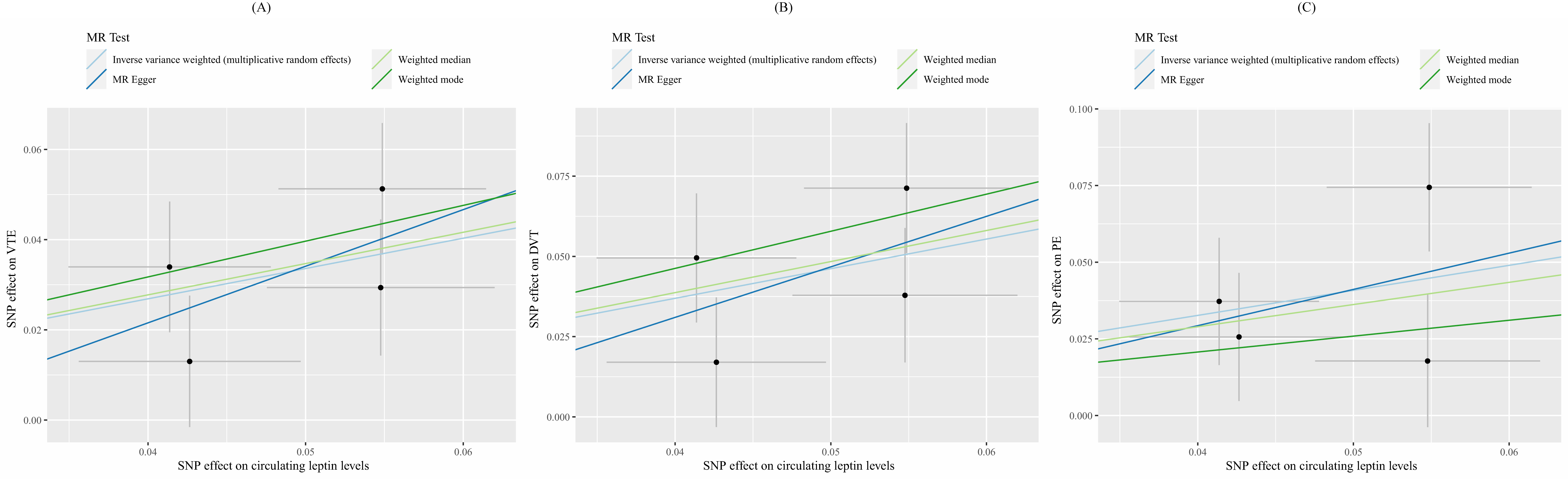

Supplement: Supplementary file 12 [file Image7.TIF]

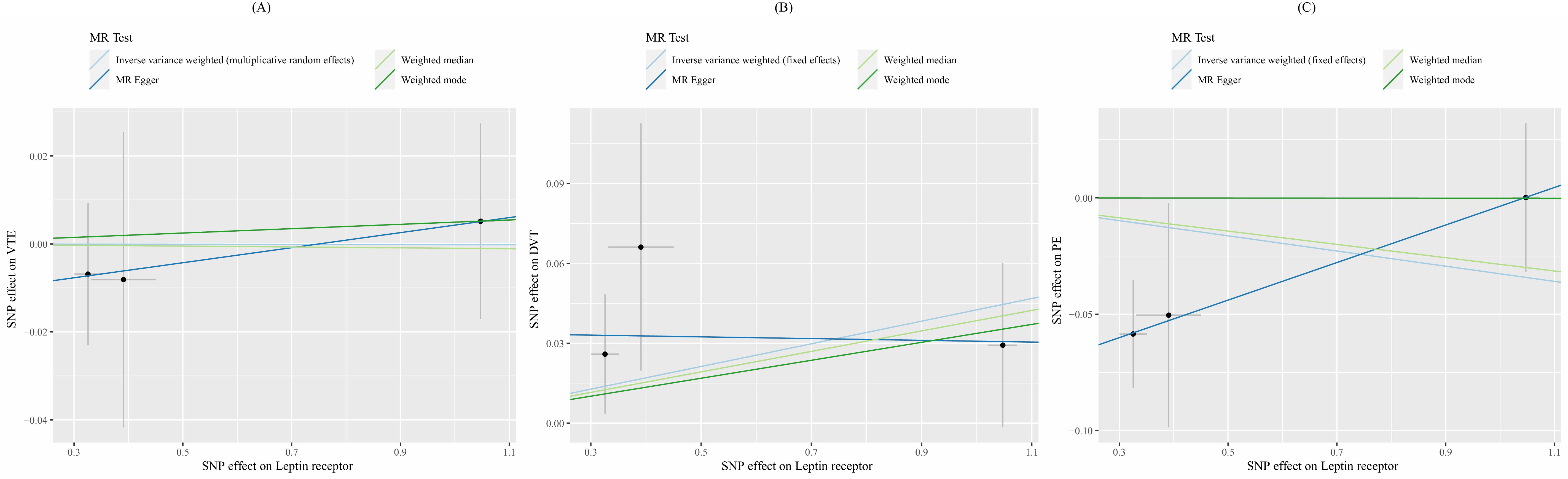

Supplement: Supplementary file 15 [file Image13.JPEG]

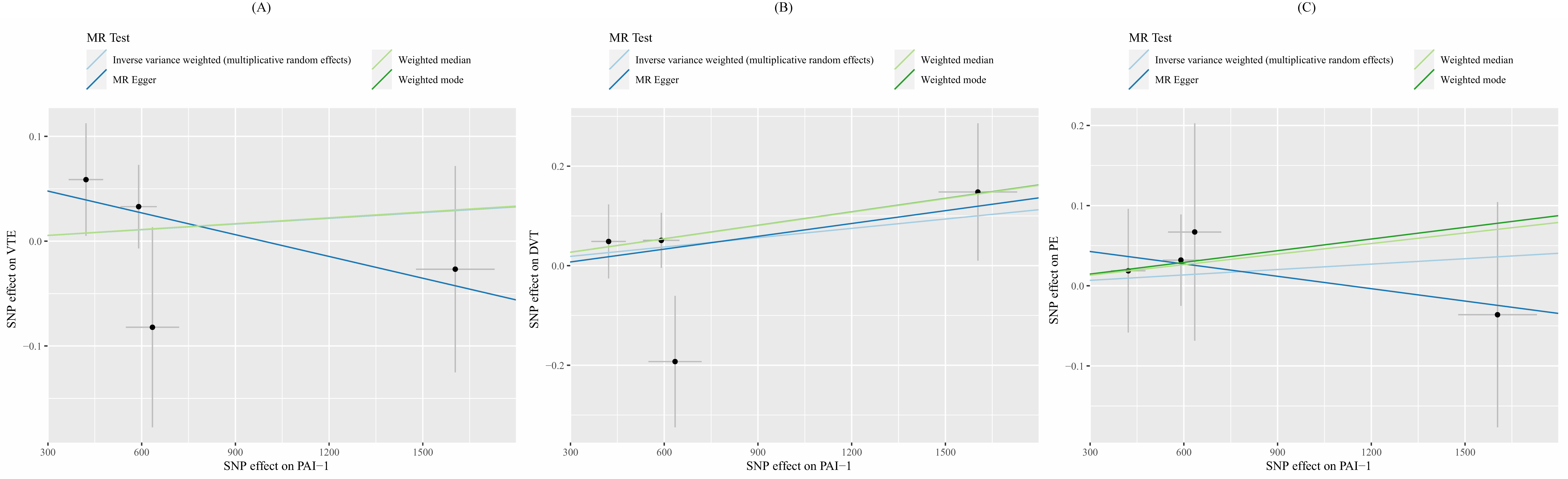

Supplement: Supplementary file 16 [file Image8.TIF]

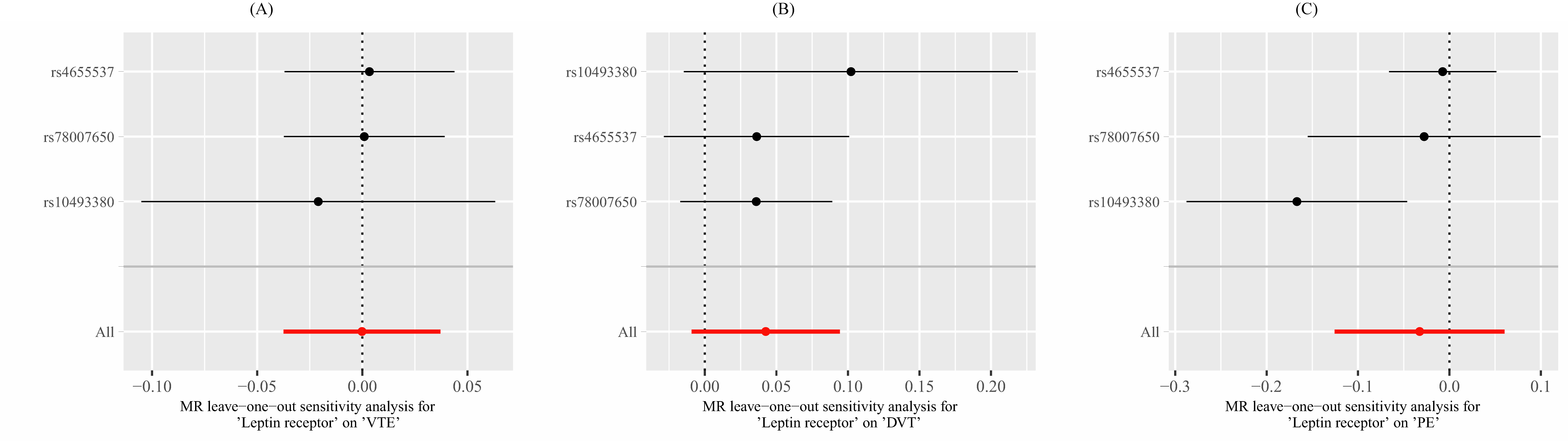

Supplement: Supplementary file 17 [file Image5.TIF]
