# Supplementary material for: Circulating adipokine concentrations and the risk of venous thromboembolism: A Mendelian randomization and mediation analysis
Source: Front Genet. 2023 Mar 28;14:1113111. doi: 10.3389/fgene.2023.1113111 (PMC10086141; doi:10.3389/fgene.2023.1113111)
Supplement: Supplementary file 13 [file Table2.DOCX]

Supplementary Material

Circulating adipokine concentrations and risk of venous thromboembolism: A Mendelian randomization and mediation analysis

Weizhong Xiao, Jian Li, Tianyuyi Feng, Long Jin *

*** Correspondence:**

Long Jin

longerg@hotmail.com

# Supplementary Data

Detail informations of SNPs included in this Mendelian randomization study.

# Supplementary Figures

# Supplementary Figure 1. Scatter plots of associations between Adiponectin and VTE (A), DVT (B), PE (C).

**Supplementary Figure 2.** Scatter plots of associations between Leptin and VTE (A), DVT (B), PE (C).

**Supplementary Figure 3.** Scatter plots of associations between PAI-1 and VTE (A), DVT (B), PE (C).

**Supplementary Figure 4.** Scatter plots of associations between MCP-1 and VTE (A), DVT (B), PE (C).

**Supplementary Figure 5.** Scatter plots of associations between Leptin receptor and VTE (A), DVT (B), PE (C).

**Supplementary Figure 6.** Scatter plots of associations between RETN and VTE (A), DVT (B), PE (C).

**Supplementary Figure 7.** Scatter plots of associations between BMI and VTE (A), DVT (B), PE (C), Leptin (D).

**Supplementary Figure 8.** Leave-one-out analysis for Adiponectin and VTE (A), DVT (B), PE (C).

**Supplementary Figure 9.** Leave-one-out analysis for Leptin and VTE (A), DVT (B), PE (C).

**Supplementary Figure 10.** Leave-one-out analysis for PAI-1 and VTE (A), DVT (B), PE (C).

**Supplementary Figure 11.** Leave-one-out analysis for MCP-1 and VTE (A), DVT (B), PE (C).

**Supplementary Figure 12.** Leave-one-out analysis for Leptin receptor and VTE (A), DVT (B), PE (C).

**Supplementary Figure 13.** Leave-one-out analysis for RETN and VTE (A), DVT (B), PE (C).

**Supplementary Figure 14.** Leave-one-out analysis for BMI and VTE (A), DVT (B), PE (C).

**Supplementary Figure 15.** Leave-one-out analysis for BMI and Leptin.
